# Rapid Diagnosis of Tuberculosis with the Xpert MTB/RIF Assay in High Burden Countries: A Cost-Effectiveness Analysis

**DOI:** 10.1371/journal.pmed.1001120

**Published:** 2011-11-08

**Authors:** Anna Vassall, Sanne van Kampen, Hojoon Sohn, Joy S. Michael, K. R. John, Saskia den Boon, J. Lucian Davis, Andrew Whitelaw, Mark P. Nicol, Maria Tarcela Gler, Anar Khaliqov, Carlos Zamudio, Mark D. Perkins, Catharina C. Boehme, Frank Cobelens

**Affiliations:** 1 Department of Global Health, and Amsterdam Institute of Global Health and Development, Academic Medical Center, Amsterdam, The Netherlands; 2 Department of Global Health and Development, London School of Hygiene & Tropical Medicine, London, United Kingdom; 3 Department of Epidemiology and Biostatistics, McGill University, Canada; 4 Christian Medical College, Vellore, India; 5 National TB Program, Vellore, India; 6 Makerere University - University of California, San Francisco (MU-UCSF) Research Collaboration, Kampala, Uganda; 7 Division of Pulmonary and Critical Care Medicine, San Francisco General Hospital, University of California, San Francisco, United States of America; 8 National Health Laboratory Service, Groote Schuur Hospital, Cape Town, South Africa; 9 Division of Medical Microbiology and Institute for Infectious Diseases and Molecular Medicine, University of Cape Town, South Africa; 10 Tropical Disease Foundation, Manila, Philippines; 11 Special Treatment Institution, Baku, Azerbaijan; 12 Instituto de Medicina Tropical Alexander von Humboldt, Universidad Peruana Cayetano Heredia, Lima, Peru; 13 Foundation for Innovative New Diagnostics (FIND), Geneva, Switzerland; Edendale Hospital, South Africa

## Abstract

**Background:**

Xpert MTB/RIF (Xpert) is a promising new rapid diagnostic technology for tuberculosis (TB) that has characteristics that suggest large-scale roll-out. However, because the test is expensive, there are concerns among TB program managers and policy makers regarding its affordability for low- and middle-income settings.

**Methods and Findings:**

We estimate the impact of the introduction of Xpert on the costs and cost-effectiveness of TB care using decision analytic modelling, comparing the introduction of Xpert to a base case of smear microscopy and clinical diagnosis in India, South Africa, and Uganda. The introduction of Xpert increases TB case finding in all three settings; from 72%–85% to 95%–99% of the cohort of individuals with suspected TB, compared to the base case. Diagnostic costs (including the costs of testing all individuals with suspected TB) also increase: from US$28–US$49 to US$133–US$146 and US$137–US$151 per TB case detected when Xpert is used “in addition to” and “as a replacement of” smear microscopy, respectively. The incremental cost effectiveness ratios (ICERs) for using Xpert “in addition to” smear microscopy, compared to the base case, range from US$41–$110 per disability adjusted life year (DALY) averted. Likewise the ICERS for using Xpert “as a replacement of” smear microscopy range from US$52–$138 per DALY averted. These ICERs are below the World Health Organization (WHO) willingness to pay threshold.

**Conclusions:**

Our results suggest that Xpert is a cost-effective method of TB diagnosis, compared to a base case of smear microscopy and clinical diagnosis of smear-negative TB in low- and middle-income settings where, with its ability to substantially increase case finding, it has important potential for improving TB diagnosis and control. The extent of cost-effectiveness gain to TB programmes from deploying Xpert is primarily dependent on current TB diagnostic practices. Further work is required during scale-up to validate these findings.

*Please see later in the article for the Editors' Summary*

## Abstract 2

A cost-effectiveness study by Frank Cobelens and colleagues reveals that Xpert MTB/RIF is a cost-effective method of tuberculosis diagnosis that is suitable for use in low- and middle-income settings.

## Editors' Summary

**Background:**

Tuberculosis (TB) is a bacterial disease that infects one-third of the world's population. The disease is caused by *Mycobacterium tuberculosis*, a bacterium that most commonly infects the lungs (known as pulmonary TB) and is transmitted from person to person when an infected individual coughs, sneezes, or talks. The symptoms of TB include chest pain, weight loss, fever, and a persistent cough that sometimes contains blood. Only 5%–10% of people who are infected with TB become sick or infectious, but people with weakened immune systems, such as individuals who are HIV-positive, are more likely to develop the disease. TB is estimated to have killed 1.7 million people in 2009 and is currently the leading cause of death among people infected with HIV.

**Why Was This Study Done?:**

Although TB can be treated with a six-month course of antibiotics, effectively diagnosing TB is not always straightforward and drug resistance is becoming an increasing problem. One of the most common and simple methods to diagnose TB is a technique called sputum smear microscopy, which involves examining matter from the lungs under a microscope for the presence of TB-causing bacteria. However, despite being cheap and relatively simple, the test does not always detect active TB (smear-negative) and cannot determine whether the TB-causing bacteria are resistant to antibiotics. The World Health Organization has recently endorsed a new rapid test, called Xpert MTB/RIF (referred to as Xpert), for the initial diagnosis of TB. The test uses DNA amplification methods to reliably and quickly detect TB and whether infecting bacteria are resistant to the antibiotic rifampicin. The new test is expensive so there are concerns that the test might not be cost-effective in low- and middle-income countries.

**What Did the Researchers Do and Find?:**

The researchers used a technique called modeling to simulate the outcome of 10,000 individuals with suspected TB as they went through a hypothetical diagnostic and treatment pathway. The model compared the costs associated with the introduction of Xpert to a base case for two different scenarios. In the base case all individuals with suspected TB had two sputum smear microscopy examinations followed by clinical diagnosis if they were smear-negative. For the different scenarios Xpert was either used in addition to the two sputum smear microscopy examinations (if the patient was smear-negative) or Xpert was used as a replacement for sputum smear microscopy for all patients. Different input parameters, based on country-specific estimates, were applied so that the model reflected the implementation of Xpert in India, South Africa, and Uganda.

In the researcher's model the introduction of Xpert increased the proportion of TB-infected patients who were correctly diagnosed with TB in any of the settings. However, the cost per TB case detected increased by approximately US$100 in both scenarios. Although the cost of detection increased significantly, the cost of treatment increased only moderately because the number of false-positive cases was reduced. For example, the percentage of treatment costs spent on false-positive diagnoses in India was predicted to fall from 22% to 4% when Xpert was used to replace sputum smear microscopy. The model was used to calculate incremental cost effectiveness ratios (ICERs—the additional cost of each disability-adjusted life year [DALY] averted) for the different scenarios of Xpert implementation in the different settings. In comparison to the base case, introducing Xpert in addition to sputum smear microscopy produced ICERs ranging from US$41 to US$110 per DALY averted, while introducing Xpert instead of sputum smear microscopy yielded ICERs ranging from US$52 to US$138 per DALY averted.

**What Do These Findings Mean?:**

The findings suggest that the implementation of Xpert in addition to, or instead of, sputum smear microscopy will be cost-effective in low- and middle-income countries. The calculated ICERs are below the World Health Organization's “willingness to pay threshold” for all settings. That is the incremental cost of each DALY averted by introduction of Xpert is below the gross domestic product per capita for each country ($1,134 for India, $5,786 South Africa, and $490 for Uganda in 2010). However, the authors note that achieving ICERs below the “willingness to pay threshold” does not necessarily mean that countries have the resources to implement the test. The researchers also note that there are limitations to their study; additional unknown costs associated with the scale-up of Xpert and some parameters, such as patient costs, were not included in the model. Although the model strongly suggests that Xpert will be cost-effective, the researchers caution that initial roll-out of Xpert should be carefully monitored and evaluated before full scale-up.

**Additional Information:**

Please access these Web sites via the online version of this summary at http://dx.doi.org/10.1371/journal.pmed.1001120.

- The World Health Organization provides information on all aspects of tuberculosis, including tuberculosis diagnostics and the Stop TB Partnership (some information is in several languages)
- The US Centers for Disease Control and Prevention has information about tuberculosis, including information on the diagnosis of tuberculosis disease
- MedlinePlus has links to further information about tuberculosis (in English and Spanish)

## Introduction

Tuberculosis (TB) control in developing countries is hampered by the inadequate care that can be delivered on the basis of diagnosis by microscopy alone—an issue that is acute in populations with a high prevalence of HIV and/or multidrug resistant (MDR)-TB. It is estimated that 1.7 million people died from TB in 2009, many of them remaining undiagnosed [1]. The Xpert MTB/RIF assay (referred to as Xpert in this article), is a real-time PCR assay that is a design-locked, all-within-cartridge test, and that has demonstrated high performance and could be deployed in a range of low- and middle-income settings [2],[3]. It has recently been endorsed by the World Health Organization (WHO) as a promising new rapid diagnostic technology that has the potential for large-scale roll-out (www.who.int/tb/laboratory). Xpert is commercially produced and sold at concessional prices. However, because the price is considerably higher than that of smear microscopy, there is a concern among TB program managers and policy makers that Xpert may not be cost-effective in low- and middle-income settings.

There is little previous research into the cost-effectiveness of TB diagnostics. A study considering a hypothetical TB diagnostic found that cost-effectiveness would be maximized by high-specificity, low-cost tests deployed in regions with poor infrastructure [4]. Other studies have examined the cost-effectiveness of culture, PCR, and novel methods for drug susceptibility testing such as line-probe assays (LPA). These studies all found that these diagnostic tests are potentially cost-effective [5]–[7]. However, because of their technical requirements, mycobacterial culture, PCR, and LPA can only be deployed in specialised laboratories. We present the first (to our knowledge) economic evaluation of the Xpert rapid test for TB. [2].

## Methods

The aim of this study was to assess whether Xpert is likely to result in an improvement of the cost-effectiveness of TB care in low- and middle-income settings. We did this by estimating the impact of Xpert on the costs and cost-effectiveness of TB care in three countries, using decision analytic modelling, comparing the introduction of Xpert to a base case of sputum microscopy and clinical diagnosis. The model's primary outcome measure is the cost per disability adjusted life year (DALY) averted.

Our model followed a cohort of 10,000 individuals suspected of having TB through the diagnostic and treatment pathway, estimating costs and health gains. In the diagnostic pathway, the TB cases among the individuals with suspected TB were either diagnosed as having TB or not, depending on the test sensitivities in the pathway. Similarly, individuals with suspected TB who were not TB cases may have been diagnosed as having TB, depending on the pathway's test specificities. A diagnosis of TB was followed by treatment. Individuals with suspected TB completed the pathway when they were either cured, failed treatment, died, or, if they had no TB from the start, remained without TB.

**Figure 1:**
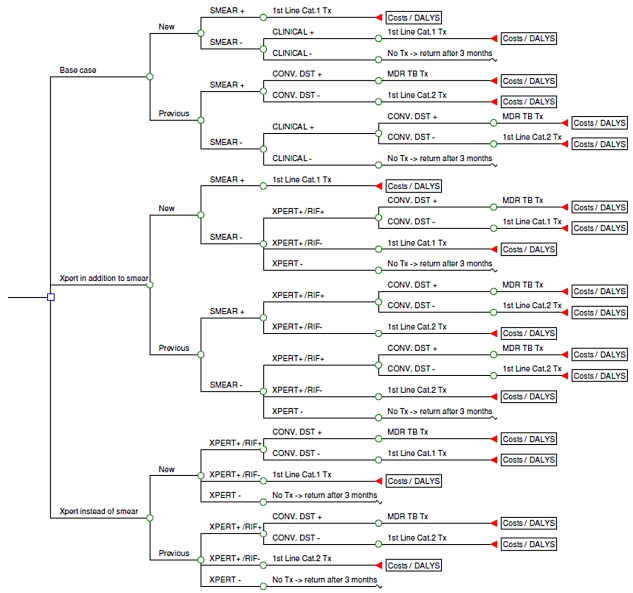
Simplified schematic of model.

Three different diagnostic scenarios are compared (Figure 1). The base case is defined as two sputum microscopy examinations followed, in smear-negative individuals with suspected TB, by clinical diagnosis that might include chest X-ray and antibiotic trial [8]. The inclusion of an antibiotic trial (empirical treatment with one or more broad-spectrum antibiotics to exclude other infectious causes of pulmonary disease) is no longer part of the WHO diagnostic strategy for HIV-infected patients. However, in the clinics participating in the demonstration study from which the diagnostic performance parameters were sourced [2], an antibiotic trial was still commonly provided during the diagnostic process as an adjunct to the treatment of smear-negative individuals with suspected TB. Antibiotic trial was therefore included in the base case; the model assumed that for each country the use of antibiotic trial and chest X-ray was proportional to the observed use in the demonstration study clinics. In comparison, two alternative pathways involving Xpert were considered: (1) two smear examinations, if negative followed by Xpert on a single sputum specimen (“in addition to”); (2) Xpert instead of smear examination: single sputum specimen tested by Xpert for all individuals with suspected TB (“replacement of”).

Each scenario included drug resistance testing of previously treated patients [9], either by conventional drug susceptibility testing (DST) or Xpert. All patients diagnosed with TB were treated using the standard WHO-recommended regimens. Patients awaiting DST results were started on first-line treatment (isoniazid [H], rifampicin [R], pyrazimamide [Z], and ethambutol [E] for 2 mo followed by HR for 4 mo for new patients, and HRZE for 3 mo with streptomycin added during the first 2 mo followed by HRE for 5 mo for patients with a history of previous TB treatment) and switched to second-line treatment when a DST result of rifampicin resistance became available. The second-line treatment regimens differed between the countries but commonly included a fluoroquinolone and an aminoglycoside (kanamycin, amikacin) or capreomycin in addition to one or more first-line drugs and ethionamode, cycloserine, and/or 4-aminosalicylic acid (PAS). If Xpert identified rifampicin resistance, this was confirmed by conventional DST or LPA as practice in the respective countries. LPA, used as a screening test on smear-positive sputum samples in South Africa, detects rifampicin resistance within 24 h by molecular methods. While awaiting this result, the patient was started on second-line treatment, but then switched to first-line treatment if resistance to rifampicin was not confirmed. TB cases that remained undiagnosed were assumed to return to the clinic after 3 mo, with 10% of undiagnosed cases becoming smear-positive within that time.

Key model input parameters are shown in Table 1 and further details can be found in Text S1. The model was parameterised for three settings: India (low HIV prevalence, low MDR prevalence), Uganda (high HIV prevalence, low MDR prevalence), and South Africa (high HIV prevalence, high MDR prevalence). In each cohort, TB cases were characterized as: (1) new or previously treated, (2) HIV-negative or HIV-positive, and (3) MDR or drug susceptible. These proportions were sourced from country reports [1],[10],[11].

**Table 1:** Model inputs: cohort composition and diagnostic parameters, by country.

Cohort Proportions and Diagnostic Parameters	India	South Africa	Uganda	Distribution	Source
**Cohort proportions**					
Smear-positive TB	0.1	0.1	0.1	Beta	Model assumption
Smear-positive TB among pulmonary TB cases, HIV-negative	0.723	0.723	0.723	Beta	Demonstration study, all sites [2]
Smear-positive TB among pulmonary TB cases, HIV-positive	0.446	0.446	0.446	Beta	Demonstration study, all sites [2]
Previous TB treatment among pulmonary TB cases	0.192	0.168	0.073	Beta	WHO [1]
Multidrug resistance, among new TB cases	0.023	0.066	0.011	Beta	WHO [10]
Multidrug resistance, among previously treated TB cases	0.172	0.245	0.117	Beta	WHO [10], survey [11]
HIV infection, among pulmonary TB cases	0.006	0.588	0.593	Beta	WHO [1]
**Diagnostic parameters**					
**Sensitivity for diagnosing pulmonary TB (SEM)**					
Xpert MTB RIF, smear-positive TB cases	0.983 (0.005)	0.983 (0.005)	0.983 (0.005)	Beta	Demonstration study, all sites [2]
Xpert MTB RIF, smear-negative TB cases, HIV-negative	0.793 (0.025)	0.793 (0.025)	0.793 (0.025)	Beta	Demonstration study, all sites [2]
Xpert MTB RIF, smear-negative cases, HIV-positive	0.718 (0.040)	0.718 (0.040)	0.718 (0.040)	Beta	Demonstration study, all sites [2]
Smear microscopy (two slides), HIV-positive	0.723 (0.015)	0.723 (0.015)	0.723 (0.015)	Beta	Demonstration study, all sites [2]
Smear microscopy (two slides), HIV-negative	0.446 (0.036)	0.446 (0.036)	0.446 (0.036)	Beta	Demonstration study, all sites [2]
Mycobacterial culture	1 (—)	1 (—)	1 (—)		Model assumption
Clinical diagnosis	0.160 (0.073)	0.209 (0.039)	0.444 (0.096)	Beta	Demonstration study [2]
Proportion culture-positive individuals with suspected TB who had chest X-ray	0.032	0.262	0.867	Beta	Demonstration study [2]
Proportion culture-positive individuals with suspected TB who had antibiotic trial	1	0.051	0.241	Beta	Demonstration study [2]
**Specificity for diagnosing pulmonary TB (SEM)**					
Xpert MTB RIF	0.990 (0.002)	0.990 (0.002)	0.990 (0.002)	Beta	Demonstration study, all sites [2]
Smear microscopy (two slides)	1 (—)	1 (—)	1 (—)		Model assumption
Mycobacterial culture	1 (—)	1 (—)	1 (—)		Model assumption
Clinical diagnosis	0.942 (0.009)	0.953 (0.007)	0.869 (0.030)	Beta	Demonstration study [2]
Proportion culture-negative individuals with suspected TB who had chest X-ray	0.037	0.059	0.790	Beta	Demonstration study [2]
Proportion culture-negative individuals with suspected TB who had antibiotic trial	1	0.009	0.887	Beta	Demonstration study [2]
**Sensitivity for detecting rifampicin-resistance (SEM)**					
Xpert MTB RIF	0.944 (0.015)	0.944 (0.015)	0.944 (0.015)	Beta	Demonstration study, all sites [2]
Conventional drug susceptibility testing	1 (—)	—	1 (—)	—	Model assumption
Line-probe assay	—	1 (—)	—	—	Model assumption
**Specificity for detecting rifampicin-resistance (SEM)**					
Xpert MTB RIF	0.983 (0.005)	0.983 (0.005)	0.983 (0.005)	Beta	Demonstration study, all sites [2]
Drug susceptibility testing	1 (—)	—	1 (—)	—	Model assumption
Line-probe assay	—	1 (—)	—	—	Model assumption
**Cost parameters** US$ 2010 (min, max)					
First-line category 1 treatment: total	227 (103, 352)	454(306, 602)	185 (146, 224)	Triangular	WHO-CHOICE [13], literature review [14]–[19]
First-line category 2 treatment: total	352 (159, 546)	998 (451, 1546)	287 (130, 445)	Triangular	WHO-CHOICE [13], literature review [14]–[19]
Cotrimoxazol preventive treatment: 1 mo	4, 50	10, 53	3, 25	Triangular	WHO-CHOICE [13]
Treatment of bacterial infection	3, 66	9, 70	2, 41	Triangular	WHO-CHOICE [13]
Chest X-ray	11 (9, 13)	16 (14, 18)	3 (2.6, 3.7)	Triangular	WHO-CHOICE [13], literature review [14]–[19]
Second-line treatment total	2,256 (1,463, 3,050)	3,492 (2,068, 4,917)	1,759 (1,285, 2,233)	Triangular	WHO-CHOICE [13], literature review [14]–[19]
**DALY parameters:** DALYs averted (min, max)					
HIV positive, sputum smear-negative	9.38 (8.62, 10.39)	10.71 (9.85, 11.90)	11.58 (10.63, 12.90)	Triangular	See Text S1
HIV negative, sputum smear-negative	13.18 (12.32, 13.96)	13.83 (12.83, 14.72)	18.65 (17.56, 19.61)	Triangular	See Text S1
HIV positive, sputum smear-positive	9.67 (8.62, 10.39)	11.03 (9.85, 11.90)	11.92 (10.63, 12.90)	Triangular	See Text S1
HIV negative, sputum smear-positive	16.43 (16.02, 16.79)	17.52 (17.05, 17.93)	22.63 (22.13, 23.07)	Triangular	See Text S1

The distribution column indicates which probability distribution was specified for each parameter in the Monte Carlo simulations. For triangular distributions the mode, upper and lower limit are given. All beta distributions have boundaries (0, 1).

SEM, standard error of the mean.

Diagnostic test performance data were sourced from a demonstration study of Xpert use in nine facilities [2]. Sensitivity and specificity parameters for all diagnostic tests and procedures were calculated taking sputum culture as the reference standard. The sensitivity and specificity of Xpert and sputum microscopy (light-emitting diode [LED]) fluorescence microscopy) was based on weighted averages across the sites. Since clinical diagnostic practice of smear negatives in the base case varied considerably between sites, site-specific data were used to estimate performance of the clinical TB diagnosis. A patient was defined as having clinically diagnosed TB if microscopy was negative, but the onset of treatment preceded the availability of the culture result.

Estimates of the economic costs of each pathway were made from a health service perspective. All costs were estimated using the ingredient costing approach. This approach identifies all the inputs (and their quantities) required to perform a test or deliver treatment and then values them to arrive at a cost per test/person treated.

Diagnostic costs were collected at the demonstration sites. These costs included all building, overhead, staff, equipment and consumables, quality control and maintenance, and calibration inputs. The resource use associated with each test was measured through observations of practice, a review of financial reporting, and interviews with staff in the Xpert demonstration sites. Resource use measurement took into account the allocation of fixed resources between Xpert and any other uses. Estimates of device and test prices, calibration, and training costs were obtained from suppliers. Costs for treatment were estimated using drugs costs from the Global Drug Facility and the MSH International Price Tracker, and unit costs for outpatient visits and hospitalisation sourced from WHO-CHOICE [12]. A review of previous costing studies was used to validate these estimates [13]–[18]. As our constructed estimates were higher than those found in our review, we took the mid-point between our estimate and the lowest estimate found in the literature. All local costs were converted using the average exchange rate for 2010 (imf.statex.imf.org). Where relevant, costs were annualised using a standard discount rate of 3% [19]. All costs are reported in 2010 US$. Treatment outcome probabilities were taken from published meta-analyses of clinical trials, cohort studies, and systematic reviews [20]–[28]. DALYs averted from patients being cured were estimated using the standard formula [19]. Further details can be found in Text S1.

Since the Xpert scenarios are most likely to be more costly and more effective than the base case, an incremental cost effectiveness ratio (ICER) was calculated to describe the additional cost for any additional DALYs averted by Xpert over the base case. This ICER was then compared to WHO's suggested country-specific willingness to pay (WTP) threshold, defined as the cost per DALY averted of each country's per capita GDP (US$1,134 for India, US$5,786 for South Africa, and US$490 for Uganda in 2010). If the ICER is below this threshold the intervention is considered cost-effective.

In the demonstration study from which our parameter estimates were sourced [2], the probability that an individual with suspected TB was a true TB case varied considerably by location; the proportion with smear-positive TB being 8.9% in India, 14.3% in South Africa, and 32.4% in Uganda. This variation probably reflects the local patterns of (self-) referral, in particular for the extremely high proportion of TB cases among the individuals with suspected TB in Uganda. Therefore to enable generalizability, we assumed a 10% proportion of smear-positive TB in individuals with suspected TB for all three countries as our point estimate with a range of 2.5% to 25% in our uncertainty and sensitivity analyses [29].

A large number of one- and two-way sensitivity analyses were conducted to assess the robustness of our model. These analyses examine the robustness of our results when one or two parameters are varied between the outer limits of their confidence intervals. We examined the sensitivity of our results to the probability that a suspect has TB or MDR-TB or has been previously treated. We examined the impact of varying treatment costs on our results. We tested for different prices of Xpert cartridge. We examined the impact of varying the proportion of individuals with suspected TB who get chest X-ray in addition to Xpert, as physicians may continue clinical diagnosis for smear-negative TB. Similarly we examined the impact of assuming that all HIV-infected individuals with suspected TB who have negative Xpert undergo the clinical diagnosis procedure, with costs based on site-specific use of chest X-rays and antibiotics, and sensitivity and specificity based on site-specific diagnostic performance of clinical diagnosis. We assessed the sensitivity of our results to the performance of the base case in three ways: (1) assuming one instead of two smears; (2) by varying the sensitivity of smear examination; and (3) by replacing the site-specific performance estimates for clinical diagnosis with estimates averaged across the three sites. Recognising that the performance of clinical diagnosis is a trade-off between sensitivity and specificity, we varied the sensitivity and specificity in opposite directions across a plausible range of values. As physicians in the demonstration study were aware that they would receive the results of sputum culture of all individuals with suspected TB, we tested for the effect of deferring treatment decisions until the availability of culture results. For each site culture was costed and assessed on the basis of current practice. We did not include a sensitivity analysis of the use of alternatives to culture such as microscopic observation drug susceptibility test (MODS) [30], as this was not practiced on site, and we found no good source of costing data. We examined the effect of reprogramming Xpert so that no resistance result is obtained.

In addition, we conducted a probabilistic sensitivity analysis (Monte Carlo simulation) to explore the effect of uncertainty across our model parameters. This analysis randomly sampled each parameter in our model simultaneously from their probability distribution (Table 1; Text S1), and repeated this 10,000 times to generate confidence intervals around our estimates of incremental cost per DALY averted.

The model and the analyses were constructed using TreeAge software. Percentage ranges in the text reflect ranges across countries unless stated otherwise.

The demonstration study was endorsed by national TB programmes of participating countries and approved by nine governing institutional review boards (IRBs). The requirement to obtain individual informed consent was waived. The costing and cost-effectiveness assessments were outlined in the study protocol reviewed by the IRBs.

## Results

The cost for the Xpert test (including all costs, such as the cartridge, equipment, salaries) ranges from US$22.63 in India to US$27.55 in Uganda, at an Xpert cartridge price of US$19.40 (including a 25% mark-up for transportation) and US$17,000 per four-module instrument (Tables 2 and 3) [2]. This cost falls to as low as US$14.93 with volume-driven price reductions. As FIND has negotiated a fixed price for Xpert, the difference in costs between sites is primarily determined by the intensity of use of the four-module instrument. Other factors also influence costs, but to a lesser extent; these include local wage levels and the room space used. A single sputum smear examination costs between US$1.13 and US$1.63. Unit costs for culture (Löwenstein–Jensen [LJ] or mycobacteria growth indicator tube [MGIT]) range from US$13.56 to US$18.95. Unit costs for tests that diagnose MDR-TB (where relevant for all first-line drugs) range from US$20.23 for LPA only to US$44.88 for MGIT and LPA.

**Table 2:** Cost of diagnostic tests at the study sites (2010 US$).

Diagnostic Test	Type of Laboratory	Costs per Test (2010 US$)		
		India	South Africa	Uganda
AFB Smear (one smear)	Peripheral/hospital	1.13	1.58	1.63
Xpert (current pricing) US$19.4 including transport	Peripheral/hospital	22.63	25.90	27.55
Xpert (volume>1.5 million/y) US$15.5 including transport	Peripheral/hospital	18.73	22.00	23.61
Xpert (volume>3.0 million/y) US$11.7 including transport	Peripheral/hospital	14.93	18.20	19.85
Culture (LJ)	Reference	13.56	—	15.45
Culture (MGIT)	Reference	—	15.24	18.95
Culture + DST (LJ)	Reference	22.33	—	23.98
Culture + DST (MGIT)	Reference	—	41.17	44.88
DST (MGIT + LPA)	Reference	—	33.01	38.82
DST (LPA), on sputum	Reference	—	20.23	21.84

**Table 3:** Cost of Xpert (current pricing) by input type (2010 US$).

Input Type	Costs per Test (2010 US$)		
	India	South Africa	Uganda
Overhead	0.18	0.88	0.40
Building space	0.02	0.08	0.12
Equipment	2.84	3.50	7.00
Staff	0.11	1.82	0.24
Reagents and chemicals	19.40	19.40	19.40
Consumables	0.07	0.22	0.38
**Total**	**22.63**	**25.90**	**27.55**

The use of Xpert substantially increases TB case finding in all three settings; from 72%–85% to 95%–99% of the TB suspect cohort (Table 4). When Xpert is deployed “as a replacement of” instead of “in addition to” smear microscopy, the number of TB cases detected is similar—while the number of MDR-TB cases detected increases substantially. When undiagnosed TB patients are assumed not to return for diagnosis, TB case detection increases from 62%–76% in the base case to 86%–94% in the Xpert scenarios.

**Table 4:** Cohort, cases detected, total cohort costs, and costs per case detected.

Country	Scenario	Cohort	*n* Individuals among the Cohort Who Have TB	Total TB Cases Detected	Percent of TB Cases Detected	Total MDR Cases Detected	Percent of MDR Cases Detected	Total Diagnostic Costs (2010 US$)	Diagnostic Cost per TB Case Detected, Excluding MDR (US$ 2010)	Additional Diagnostic Cost per MDR Case Detected (2010 US$)	Treatment Costs (2010 US$)	Treatment Costs Percent of Total Cohort
India	Base case	Tuberculosis (MDR)	72	59	82	38	52	1,077	—	—	89,223	19
		Tuberculosis (no MDR)	1,318	1,079	82	—	—	8,412	—	—	268,122	59
		No tuberculosis	8,611	—	—	—	—	46,106	—	—	100,759	**22**
		**Total**	**10,000**	**1,138**	**82**	**38**	**—**	**55,595**	**49**	**165**	**458,103**	100
	In addition to smear	Tuberculosis (MDR)	72	71	99	49	68	2,335	—	—	115,932	25
		Tuberculosis (no MDR)	1,318	1,300	99	—	—	13,831	—	—	325,381	70
		No tuberculosis	8,611	—	—	—	—	184,298	—	—	22,414	**5**
		**Total**	**10,000**	**1,371**	**99**	**49**		**200,464**	**146**	**116**	**463,727**	100
	Replacement of smear	Tuberculosis (MDR)	72	71	99	67	93	3,038	—	—	151,603	30
		Tuberculosis (no MDR)	1,318	1,298	99	—	—	28,986	—	—	328,669	65
		No tuberculosis	8,611	—	—	—	—	174,538	—	—	22,414	**4**
		**Total**	**10,000**	**1,369**	**99**	**67**		**206,562**	**151**	**24**	**502,687**	100
South Africa	Base case	Tuberculosis (MDR)	184	131	72	56	31	2,345	—	—	230,989	22
		Tuberculosis (no MDR)	1,729	1,237	72	—	—	13,772	—	—	659,365	63
		No tuberculosis	8,087	—	—	—	—	22,014	—	—	156,213	**15**
		**Total**	**10,000**	**1,368**	**72**	**56**		**38,131**	**28**	**86**	**1,046,567**	100
	In addition to smear	Tuberculosis (MDR)	184	175	95	112	61	7,131	—	—	423,146	31
		Tuberculosis (no MDR)	1,729	1,649	95	—	—	30,341	—	—	882,010	65
		No tuberculosis	8,087	—	—	—	—	205,858	—	—	45,788	**3**
		**Total**	**10,000**	**1,824**	**95**	**112**		**243,331**	**133**	**57**	**1,350,945**	100
	Replacement of smear	Tuberculosis (MDR)	184	175	95	165	90	9,504	—	—	583,064	39
		Tuberculosis (no MDR)	1,729	1,645	95	—	—	46,866	—	—	880,190	58
		No tuberculosis	8,087	—	—	—	—	193,053	—	—	45,788	**3**
		**Total**	**10,000**	**1,820**	**95**	**165**		**249,423**	**137**	**30**	**1,509,043**	100
Uganda	Base case	Tuberculosis (MDR)	36	30	85	14	38	499	—	—	26,422	5
		Tuberculosis (no MDR)	1,882	1,594	85	—	—	11,282	—	—	282,928	59
		No tuberculosis	8,082	—	—	—	—	51,565	—	—	171,803	**36**
		**Total**	**10,000**	**1,625**	**85**	**14**		**63,345**	**39**	**163**	**481,154**	100
	In addition to smear	Tuberculosis (MDR)	36	34	95	22	63	1,392	—	—	41,123	11
		Tuberculosis (no MDR)	1,882	1,794	95	—	—	34,694	—	—	320,685	85
		No tuberculosis	8,082	—	—	—	—	230,369	—	—	14,908	**4**
		**Total**	**10,000**	**1,828**	**95**	**22**		**266,455**	**146**	**124**	**376,717**	100
	Replacement of smear	Tuberculosis (MDR)	36	34	95	32	90	1,849	—	—	56,488	14
		Tuberculosis (no MDR)	1,882	1,790	95	—	—	57,204	—	—	322,502	82
		No tuberculosis	8,082	—	—	—	—	217,185	—	—	14,908	**4**
		**Total**	**10,000**	**1,824**	**95**	**32**		**276,238**	**151**	**27**	**393,899**	100

The diagnostic cost (including the costs of testing all individuals with suspected TB) per TB case detected is US$28–US$49 for the base case and increases significantly to US$133–US$146 and US$137–US$151 when Xpert is used “in addition to” and “as a replacement of” smear microscopy, respectively, depending on the setting (Table 4). The resulting change in treatment costs is more moderate, due to a reduction in the numbers of false positives in the base case from clinical diagnosis. For example, in India, the percentage of treatment costs spent on false-positive diagnoses falls from 22% to 4% when Xpert is used “as a replacement of” smear microscopy in comparison to the base case.

ICERs for each Xpert scenario are presented in Table 5. The mean ICER for using Xpert “in addition to” smear microscopy compared to the base case ranges from US$41 to US$110 per DALY averted depending on the setting. The mean ICER for using Xpert “as a replacement of” smear microscopy ranges from US$52 to US$138 per DALY averted. The mean ICER for using Xpert as “a replacement of” smear microscopy compared to using Xpert “in addition to” smear microscopy ranges between US$343 and US$650. This higher ICER is due to the fact that the effectiveness gain from using Xpert as “replacement of smear microscopy” is derived from additional MDR-TB cases detected, and the cost-effectiveness of treating MDR-TB is lower than that for drug-susceptible TB. All the ICERs found are well below the WTP threshold.

**Table 5:** Cost per DALY (US$ 2010).

Country	Scenario	Total Cost	Total DALYS	Cost per DALY	ICER Compared to Base Case, Mean	Monte Carlo Simulation ICER, Median (2.5, 97.5)	ICER Compared to in Addition to, Mean	Monte Carlo Simulation ICER, Median (2.5, 97.5)
India	Base case	513,698	17,133	30	—	—	—	—
	In addition to smear	664,191	19,887	33	55	54 (40, 70)	—	—
	Replacement of smear	709,248	20,019	35	68	68 (51, 87)	343	361 (239, 578)
South Africa	Base case	1,084,698	15,805	69	—	—	—	—
	In addition to smear	1,594,276	20,420	78	110	109 (88, 133)	—	—
	Replacement of smear	1,758,467	20,702	85	138	136 (105, 172)	582	594 (353, 956)
Uganda	Base case	544,499	22,182	25	—	—	—	—
	In addition to smear	643,172	24,570	26	41	34 (−3, 69)	—	—
	Replacement of smear	670,137	24,611	27	52	37 (0, 73)	650	276 (−1895, 2,406)

**Figure 2:**
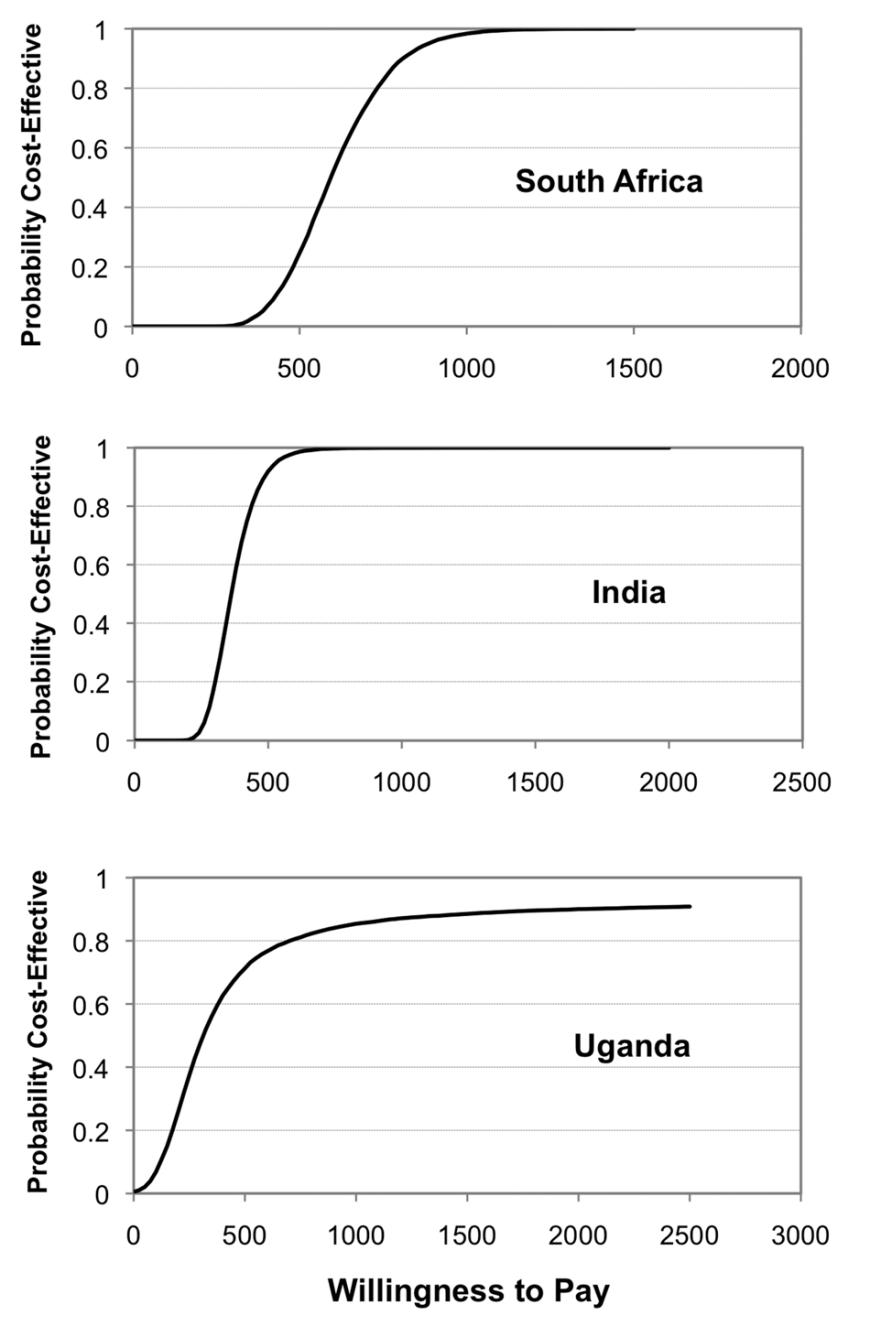
Cost-effectiveness acceptability curves. ICER “replacement of smear” compared with “in addition to smear.”

The results of the probabilistic sensitivity analysis (Monte Carlo simulation) are also shown in Table 5. Aside from the replacement of smear microscopy in Uganda all estimates remain cost-effective. Figure 2 provides an illustration of the cost-effectiveness of Xpert deployed as “a replacement of” smear microscopy in comparison to the “in addition to” scenario for a range of WTP thresholds. This graph, known as an acceptability curve, shows that if the WTP is US$490 in Uganda, there is around a 75% probability that Xpert as a replacement of smear is cost-effective when compared to the “in addition to” scenario.

**Figure 3:**
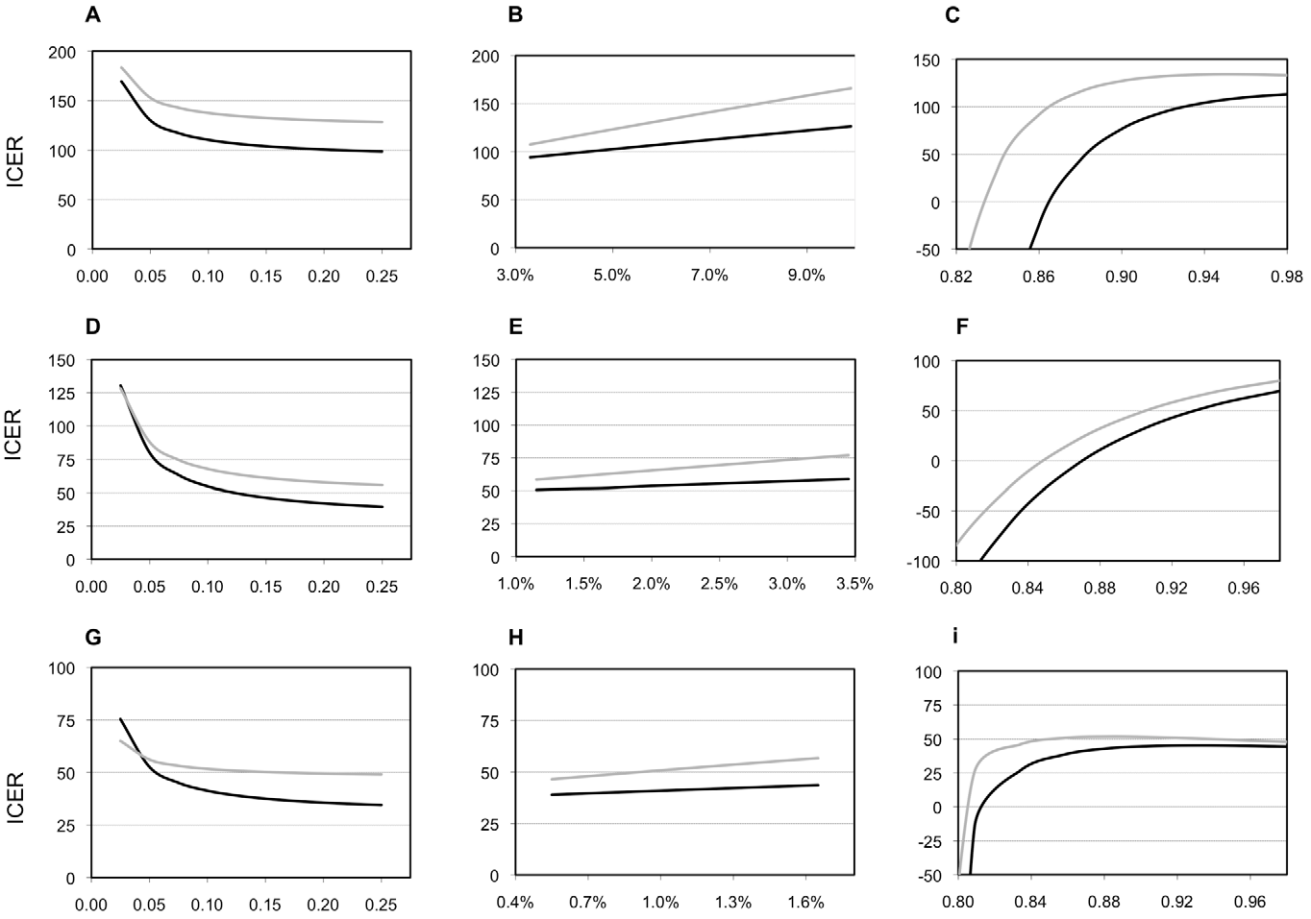
Selected sensitivity analyses. Sensitivity of the model for the prevalence of tuberculosis, for the prevalence of multidrug-resistant tuberculosis, and for the accuracy of clinical diagnosis. Patterns of ICERs in 2010 US$ for varying the proportion of individuals with suspected TB in the cohort who have smear-positive TB (A, D, G); for varying the proportion of new patients with TB who have multidrug-resistant TB (MDR-TB, B, E, H); and for varying the specificity of the clinical diagnosis of TB in the base case (C, F, I). (A, B, and C), South Africa; (E,D, and F), India; (G, H, and I), Uganda. Black lines, Xpert assay in addition to sputum smear examination; grey lines, Xpert assay as replacement of sputum smear examination. The proportion of individuals with suspected TB in the cohort who have smear-negative TB varies along with the proportion of individuals with suspected TB in the cohort who have smear-positive TB in a linear manner, depending on the HIV-infection prevalence (A, D, G; see Table 1 and Text S1). Similarly, the proportion of previously treated patients with TB who have MDR-TB varies linearly with the proportion of new patients with TB who have MDR-TB (B, E, H; see Table 1 and Text S1). The sensitivity of clinical diagnosis in the base case varies inversely with the specificity (range, 8%–80%; C, F, I).

Nearly all of our one- and two-way sensitivity analyses did not increase the ICER compared to the base case of either Xpert scenario above the WTP threshold (Table 6). Figure 3 shows ICER variation when parameters for the suspect population and the performance of the base case change. Varying the true proportion of those with TB and MDR-TB in the cohort has little effect on our results, although Xpert ICERs substantially worsen when the proportion of smear-positive TB cases becomes 5% or less (translating into 7%–9% with any type of TB). Varying assumptions on the performance of the base case alters ICERs substantially. Increasing the sensitivity of smear examination reduces the cost-effectiveness of Xpert, but not below the WTP threshold. If clinical diagnosis has a higher specificity and lower sensitivity than in our study sites, Xpert ICERs worsen, but also remain below the WTP threshold. But, if clinical diagnosis has a lower specificity and higher sensitivity than in our study sites, ICERs for Xpert substantially improve. Adding chest X-ray for 50% of the individuals with suspected TB tested by Xpert has limited impact on the cost-effectiveness of Xpert. Adding clinical diagnosis for all HIV-positive individuals with suspected TB with a negative Xpert result has no or limited effect in India and South Africa, but doubles ICERs for Xpert in Uganda (although not above the WTP threshold). This reflects differences in HIV prevalence as well as relatively high cost and low specificity of clinical diagnosis in Uganda owing to more extensive use of X-ray. Incorporating the cost of culture and increasing the proportion of TB diagnosis based on the culture result, has a mixed effect. Xpert remains cost-effective up until the point where 40%–70% of patients receive a culture-based diagnosis. Above proportions of 50%–90%, the base case becomes more effective. If however, culture performance is less than 100%, the base case does not become more effective than the Xpert-based scenarios until nearly 100% of patients receive a culture-based diagnosis (unpublished data).

**Table 6:** Costs per DALY 2010 US$: sensitivity analyses.

Assumption	ICER Compared to:	India			South Africa			Uganda		
		Base Case	In Addition to Smear	Replacement of Smear	Base Case	In Addition to Smear	Replacement of Smear	Base Case	In Addition to Smear	Replacement of Smear
Primary estimate	Base case	—	55	68	—	110	138	—	41	52
	In addition to smear	—		343	—	—	582	—	—	650
Reprogrammed test: no signal MDR	Base case	—	50	51	—	87	86	—	37	40
	In addition to smear	—		107	—	—	NA	—	—	289
Clinical diagnosis performance pooled across countries	Base case	—	62	78	—	89	121	—	53	58
	In addition to smear	—		342	—	—	582	—	—	650
Proportion retreatment doubles	Base case	—	115	119	—	209	220	—	67	73
	In addition to smear	—		170	—	—	334	—	—	200
Cartridge cost reduces to US$11.70	Base case	—	42	54	—	102	129	—	26	36
	In addition to smear	—	—	318	—		570	—	—	561
50% of individuals with suspected TB have X-ray added to Xpert	Base case	—	73	87	—	126	154	—	47	58
	In addition to smear	—	—	378	—	—	606	—	—	686
HIV-infected individuals with suspected TB have clinical diagnosis when Xpert is negative	Base case	—	55	68	—	132	157	—	82	90
	In addition to smear	—		343	—		610	—		706
Undiagnosed patients with TB do not return for diagnosis	Base case	—	50	67	—	109	138	—	33	43
	In addition to smear	—	—	Dominated by in addition to scenario	—	—	1,442	—	—	Dominated by in addition to scenario
Single smear examination in base case	Base case	—	48	58	—	105	130	—	40	48
	In addition to smear	—	—	343	—	—	582	—	—	650
60% of case receive culture diagnosis	Base case	—	Dominates base case	Dominates base case	—	67	311	—	Base case more cost-effective	Base case more cost-effective
	In addition to smear	—	—	343	—	—	582	—	—	650
Sensitivity of smear examination increase by 15%	Base case	—	106	130	—	131	165	—	59	74
	In addition to smear	—	—	343	—	—	582	—	—	650

NA, not available.

## Discussion

Our results suggest that Xpert is likely to be more cost-effective than a base case of smear microscopy and clinical diagnosis of smear-negative TB. The extent and type of cost-effectiveness gain from deploying Xpert is dependent on a number of different setting-specific factors. First and foremost of these factors is the performance of current TB diagnostic practice. Where the sensitivity of current practice is low, but specificity high, Xpert has a substantial impact on effectiveness. Where the sensitivity of current practice is high, but specificity low, Xpert will lower treatment costs by reducing the number of false positives. This latter effect is illustrated by the case of Uganda, where the model predicts a reduction in the treatment costs of false positives from US$171,803 to US$14,908, contributing to the overall reduction in treatment costs.

Other factors that are likely to determine the extent of cost-effectiveness gain include the proportion of those co-infected with HIV and the proportion of those with MDR-TB, and the numbers of true TB cases in the suspect population. However, our results show that increasing proportions of HIV in the suspect population will not always reduce the ICER of Xpert (Figure 3). This finding is counter-intuitive. One would expect the cost-effectiveness of a diagnostic test that diagnoses smear-negative TB to improve with increases in HIV prevalence. However, as the proportion of individuals co-infected with HIV in the suspect population increases, so the sensitivity of Xpert decreases. Depending on the relative costs and performance of the base case, this counter-effect means that the relationship between HIV prevalence and Xpert's cost-effectiveness is weaker than anticipated.

Nor can we conclude on the direction of the relationship between cost-effectiveness gain and the level of prevalence of MDR-TB in the suspect population at this time. Our model demonstrates that when transmission effects are excluded, the ICER of Xpert increases as the MDR-TB prevalence increases (Figure 3). This result occurs because although the effectiveness of Xpert increases with a higher MDR-TB prevalence, the ICER of treating MDR-TB is higher than that of drug susceptible TB, thus countering the gain from increased effectiveness.

Unsurprisingly, we also find that higher proportions of TB cases in the suspect population improve the cost-effectiveness of Xpert. The cost per TB case detected will also decrease with increases in TB prevalence. As TB programmes already fund elements of the base case, cost-effectiveness may therefore be initially improved by using existing diagnostic tools, such as X-ray and clinical scores, to screen the TB suspect population prior to Xpert. In the longer run, however, the expansion of X-ray as a permanent approach for suspect screening is unlikely to be cost-effective, and further work examining alternative screening approaches may be required. Moreover, different approaches are likely to be adopted for specific suspect populations, most notably those already known to be HIV infected, those who have already failed treatment, and those at a high risk of MDR-TB. We therefore recommend that further work is conducted to explore the impact on cost-effectiveness of different algorithms when Xpert is applied to more limited suspect groups.

A number of factors limit our analysis. Firstly, the assumption of no transmission effects or additional mortality benefit from early diagnosis is a conservative approach and will underestimate the cost-effectiveness of Xpert—particularly where the introduction of Xpert is likely to increase the numbers of drug-resistant patients who are appropriately and rapidly treated. Likewise, we do not factor in patient costs. A full societal evaluation would make all options less cost-effective, but Xpert is likely to fare better than alternatives, as it requires fewer patient visits. In addition, if Xpert can achieve earlier diagnosis, substantial reductions in patient costs prior to treatment may be achieved [31]. The reference standard for the test performance parameters in our model did not include culture-negative TB based on response to treatment, because this diagnostic category will include cases with no TB or extra-pulmonary TB that cannot be diagnosed by sputum-based tests. This situation may have lead to overestimation of the sensitivity and underestimation of the specificity of Xpert. Owing to lack of evidence, we only included one repeat visit for false negatives in our model, to capture those who quickly progress to smear-positive TB. This number may be insufficient and miss both the additional costs and effectiveness of further repeated visits. On the other hand, our assumption that 100% of false negatives still alive and with TB after 3 mo have a repeat visit may be an overestimation, thereby inflating ICERs for the Xpert scenarios. We assumed that a negative Xpert result does not lead to further TB diagnostic procedures. This assumption may not be true in practice, in particular not for HIV-infected individuals with suspected TB [32]. Our sensitivity analyses show that adding clinical diagnostic procedures for this group can substantially reduce cost-effectiveness of Xpert when HIV prevalence is high and X-ray is used extensively. Also because of the lack of data, we have not included a high MDR-TB, but low HIV-prevalence setting. This lack of data restricts our ability to generalise findings to all low- and middle-income settings, particularly the former Soviet states, where this epidemiological pattern is common in suspect populations. Finally, our sensitivity analysis demonstrates that Xpert may not be cost-effective when compared to a base case in which a high proportion of smear-negative TB cases are diagnosed by culture. However, this result is based on our assumption that culture performs at 100% sensitivity and specificity. In addition, we did not include costs of specimen transport, increased risk of false-negative cultures or contamination, reduced sensitivity when only one specimen is cultured, and possible delay effects on mortality and patient drop out. All these simplifications will inflate the cost-effectiveness of a base case that includes culture.

As is standard practice, we determine cost-effectiveness in comparison to the WHO WTP threshold. Unfortunately, achieving this threshold does not mean that the resources are available in low- and middle-income countries, merely that Xpert should be afforded [33]. In reality, resourcing for tuberculosis services in low- and middle-income countries is extremely constrained. Countries may therefore need to prioritise. In terms of priorities, suspect populations with a high likelihood of TB, particularly in settings with high HIV and MDR-TB prevalence, are an obvious choice. However, our findings illustrate that it is also important to balance these factors with the current performance of the existing diagnostic pathway. Countries or areas that have the weakest performance in terms of diagnosing smear-negative cases may benefit the most, even when they have relatively low levels of MDR-TB and HIV; although additional investment may be required to strengthen aspects of the health system to ensure that Xpert can be implemented successfully. Funding Xpert may also mean that scarce resources are not made available to other equally deserving areas. Care must therefore be exercised to take into account the broader tuberculosis control and health system needs of any particular setting when funding Xpert.

Our model is robust given the current evidence and data available. However, key data in this area—particularly on the characteristics of TB suspect populations, the feasibility of implementing Xpert at scale, and the extent to which clinicians allow diagnostic test results to influence treatment decisions—remain scarce. Moreover, it is likely that there will be costs associated with Xpert scale-up that we cannot predict at this point. Although our model strongly suggests that Xpert will be cost-effective in a wide variety of settings, Xpert scale-up will substantially increase TB diagnostic costs. Given this increase, and the current data paucity, we recommend careful monitoring and evaluation of initial roll-out before full scale-up. Funding should be provided for implementation studies, including pragmatic trials, in a number of countries to accelerate this process. As we did not assess cost-effectiveness in a setting with high MDR but low HIV prevalence, we also recommend additional economic modelling studies before embarking on roll-out in these settings, taking into consideration operational factors that may affect outcomes such as patient drop-out and physician behavior [34]. Finally, although Xpert is a highly promising technology, there is still room for improvement in TB diagnostics. Xpert has incomplete sensitivity for smear-negative TB and rifampicin resistance and does not detect resistance to isoniazid and other drugs. Other promising tests, such as microscopic observation drug susceptibility test (MODS) [35], should be evaluated for their cost-effectiveness, including comparisons with Xpert. Our finding should not discourage investment in other promising new TB diagnostic technologies, particularly those that further improve the diagnostic sensitivity and detection of wider forms of drug resistance and can be implemented at peripheral health care level at low cost.

### Conclusion

Despite the fact that there is considerable concern from policy makers about the costs and affordability of new diagnostic technologies in low- and middle-income countries, our results suggest that Xpert is likely to be a highly cost-effective investment. If demonstrated test performance is maintained at scale, Xpert has the potential to substantially increase TB case detection. Moreover, in the settings modelled, TB treatment costs are not predicted to substantially increase with the introduction of Xpert; instead, treatment is likely to be switched from those who do not benefit from treatment, to those who do. Our results suggest that funding should be provided to initiate the roll-out of Xpert in low- and middle-income countries, as a promising means of enabling access to effective treatment for all those with the disease. We recommend, however, that this roll-out is carefully evaluated to validate our results before full scale-up—to ensure that Xpert implementation is done in a way that does not negatively impact TB programmes, their funding, and the health systems that support them.

## Supporting Information

Text S1 Details of model assumptions, test turnaround times (Table S[A]), treatment outcome probabilities (Table S[B]), probabilities of death and spontaneous recovery with false-negative tuberculosis diagnosis (Table S[C]), and variables used in the DALY calculations (Table S[D]).
(DOC)

## Abbreviations

DALY: disability adjusted life year
DST: drug susceptibility testing
ICER: incremental cost effectiveness ratio
LJ: Lowenstein–Jensen
LPA: line probe assay
MDR: multidrug resistant
MGIT: mycobacteria growth indicator tube
TB: tuberculosis
WTP: willingness to pay
Xpert: Xpert MTB/RIF
