# Supplementary material for: Rapid Diagnosis of Tuberculosis with the Xpert MTB/RIF Assay in High Burden Countries: A Cost-Effectiveness Analysis
Source: PLoS Med. 2011 Nov 8;8(11):e1001120. doi: 10.1371/journal.pmed.1001120 (PMC3210757; doi:10.1371/journal.pmed.1001120)
Supplement: Text S1 — Details of model assumptions, test turnaround times (Table S[A]), treatment outcome probabilities (Table S[B]), probabilities of death and spontaneous recovery with false-negative tuberculosis diagnosis (Table S[C]), and variables used in the DALY calculations (Table S[D]). (DOC) [file pmed.1001120.s001.doc]

**Text S1**

**Supporting information**

**Vassall et al. The rapid diagnosis of tuberculosis with Xpert MTB-RIF assay in high burden countries: a cost-effectiveness analysis**

The model makes the following key assumptions in addition to those already mentioned in the published text:

1. *With regard to the definition of the cohorts of patients suspected of pulmonary tuberculosis (“TB suspects”):*

- At baseline, the ratio of smear-negative to smear-positive TB cases among the TB suspects is fixed within two strata of HIV status (negative, positive) and derived from the demonstration study by pooling the proportions of culture-positive patients who are smear-positive across the six study sites.
- The HIV prevalence among the TB suspects is derived from the HIV prevalence among TB patients as sourced from country reports or studies.
- The probability of MDR-TB is independent of sputum smear status, as well as of HIV status.

1. *With regard to the diagnostic scenarios:*

- The culture result, as observed in the demonstration study, is taken as the reference standard. Patients who have negative test results (sputum smear examination, clinical diagnosis, Xpert MTB/RIF) but positive culture results are considered false-negatives. Patients who have positive test results (sputum smear examination, clinical diagnosis, Xpert MTB/RIF) but negative culture results are considered false-positives.
- The clinical diagnosis of smear-negative TB (clinical evaluation, chest X-ray and antibiotic trial) is a single diagnostic procedure with fixed test characteristics, but the cost inputs reflect the observed utilization of chest X-ray and antibiotic trial in the three countries for which the models are run, separately for patients with negative and for patients with positive culture results.
- The method of drug susceptibility testing, as used in the base case and for confirmation of rifampicin resistance detected by the Xpert MTB/RIF is as is currently used routinely in the countries for which the models were run: solid media culture followed by solid media susceptibility testing in India and Uganda, and line-probe assay directly from sputum (smear-positive patients) or on a liquid medium culture isolate (smear-negative patients) in South Africa.
- In the Xpert MTB/RIF ‘in addition to smear’ scenario, Xpert MTB/RIF is used to test for rifampicin resistance in previously treated patients with positive smear examinations.
- Assumed turnaround times for the various test procedures are as mentioned in Table S1.

1. *With regard to TB treatment:*

- Treatment outcomes used in the model are listed in Table S2.
- Treatment outcomes are similar for smear-positive and smear-negative pulmonary TB.
- Relapses do not occur.
- Patients awaiting drug susceptibility testing results are started on standard category 1 or 2 treatment depending on their treatment history, and are switched to second-line treatment if, and as soon as, resistance to rifampicin is shown.
- Patients tested resistant to rifampicin by Xpert MTB/RIF and awaiting drug susceptibility testing results for confirmation are started on second-line treatment, and are switched to standard category 1 or 2 treatment depending on their treatment history if, and as soon as, an susceptibiltiy to rifampicin is shown.
- Mortality rates during an aborted treatment episode (i.e. before switching to another regimen) are proportional to the mortality rates for the specified treatment regimen given for the specified TB category and resistance pattern, assuming a uniform mortality rate over time.
- Treating patients with MDR or rifampicin resistance with first-line regimens while awaiting susceptibility results has no effect on the outcomes of subsequent second-line treatment.

*4. With regard to treatment delay:*

- No delays are assumed, except in patients with TB who have remained undiagnosed (false-negatives) and, while on initial treatment, while awaiting drug susceptibility results (see above). The delay for false-negatives until their next diagnostic visit is 3 months. If again false-negative after this visit, the patient is assumed to remain undiagnosed.
- True negative patients (i.e. who have no TB and were not diagnosed with TB) do not return for diagnosis.
- False-negative patients returning for diagnosis are assumed to have rates of mortality and spontaneous recovery as mentioned in Table S3. These rates are dependent on HIV status but not on smear status. Patients spontaneously recovered from TB do also not return.
- Of the false-negative patients with TB returning for diagnosis who were initially smear-negative, 10% have become smear-positive by the time they return. Procedures and test performance on the return visit are similar as for the initial diagnostic visit.

1. *With regard to costs*

- The following regimens were used to estimate drugs costs:
- First line: 2HRZE/ 4HR
- Second line: 2HRZES/1 HRZE/5HRE
- MDR-TB:

**India**: Daily for 6 months: ofloxacin 800mg, cycloserin 750mg, ethionamide 750mg, pyridoxine 100mg, ethambutol 200mg, ethambutol+hydrochloride 800mg, pyrazinamide 1500mg, kanamycin injection (6 times a week) 1000mg. Daily for 18 months: ofloxacin 800mg, ethionamide 750mg, ethambutol 200mg, cycloserin 750mg.

**South Africa/ Uganda:** Daily for 4 months: kanamycin 1000mg, ethionamide 1000mg, pyranazinamade 1600mg, ofloxacin 800 mg OR ciprofloxacin 1500mg, Ethambutal 1200mg or cycloserin 1000mg (all maximum doses). Daily for 12-18 months: ethionamide 500mg, ofloxacin 800mg or ciproflaxin 1500mg, ethambutal 1200mg or cycloserin 800mg.

1. *With regard to DALYs*

- YLLs averted were calculated using local age-specific life expectancy minus the median age at death from TB. This approach implicitly assumes that a reduction in mortality from TB is not substantial enough to significantly affect average local life expectancy. The median age of death was constructed using site specific median ages and rates of death and self-cure associated with missed and delayed diagnoses from the literature [1] [2][3][4]. Country-wide age-specific life expectancies were used to estimate local age-specific life expectancy for HIV-negative patients. For those who were HIV-positive a median survival time from a previously published Markov model for HIV+ patients (on ART) was applied in all settings.
- YLDs were calculated for the period of the diagnostic and treatment pathway. Disability weights for those living with TB and/or HIV were sourced from Global Burden of Disease, 2004 update.

**References**

1. Dye C, Garnett GP, Sleeman K, Williams BG (1998) Prospects for worldwide tuberculosis control under the WHO DOTS strategy. Directly observed short-course therapy. Lancet 352: 1886-1891.

2. Dye C, Williams BG (2000) Criteria for the control of drug-resistant tuberculosis. Proc. Natl. Acad. Sci. U.S.A 97: 8180-8185. doi:10.1073/pnas.140102797

3. Salomon JA, Lloyd-Smith JO, Getz WM, Resch S, Sánchez MS, et al. (2006) Prospects for advancing tuberculosis control efforts through novel therapies. PLoS Med 3: e273. doi:10.1371/journal.pmed.0030273

4. Abu-Raddad LJ, Sabatelli L, Achterberg JT, Sugimoto JD, Longini IM, et al. (2009) Epidemiological benefits of more-effective tuberculosis vaccines, drugs, and diagnostics. Proc. Natl. Acad. Sci. U.S.A 106: 13980-13985. doi:10.1073/pnas.0901720106

5. Lew W, Pai M, Oxlade O, Martin D, Menzies D (2008) Initial drug resistance and tuberculosis treatment outcomes: systematic review and meta-analysis. Ann. Intern. Med 149: 123-134.

6. Nathanson E, Lambregts-van Weezenbeek C, Rich ML, Gupta R, Bayona J, et al. (2006) Multidrug-resistant tuberculosis management in resource-limited settings. Emerging Infect. Dis 12: 1389-1397.

7. Menzies D, Benedetti A, Paydar A, Royce S, Madhukar P, et al. (2009) Standardized treatment of active tuberculosis in patients with previous treatment and/or with mono-resistance to isoniazid: a systematic review and meta-analysis. PLoS Med 6: e1000150.

8. Espinal MA, Kim SJ, Suarez PG, Kam KM, Khomenko AG, et al. (2000) Standard short-course chemotherapy for drug-resistant tuberculosis: treatment outcomes in 6 countries. JAMA 283: 2537-2545.

9. Akksilp S, Karnkawinpong O, Wattanaamornkiat W, Viriyakitja D, Monkongdee P, et al. (2007) Antiretroviral therapy during tuberculosis treatment and marked reduction in death rate of HIV-infected patients, Thailand. Emerging Infect. Dis 13: 1001-1007.

10. Varma JK, Nateniyom S, Akksilp S, Mankatittham W, Sirinak C, et al. (2009) HIV care and treatment factors associated with improved survival during TB treatment in Thailand: an observational study. BMC Infect. Dis 9: 42. doi:10.1186/1471-2334-9-42

11. Abdool Karim SS, Naidoo K, Grobler A, Padayatchi N, Baxter C, et al. (2010) Timing of initiation of antiretroviral drugs during tuberculosis therapy. N. Engl. J. Med 362: 697-706. doi:10.1056/NEJMoa0905848

12. Wells CD, Cegielski JP, Nelson LJ, Laserson KF, Holtz TH, et al. (2007) HIV infection and multidrug-resistant tuberculosis: the perfect storm. J. Infect. Dis 196 Suppl 1: S86-107. doi:10.1086/518665

13. Seung KJ, Omatayo DB, Keshavjee S, Furin JJ, Farmer PE, et al. (2009) Early outcomes of MDR-TB treatment in a high HIV-prevalence setting in Southern Africa. PLoS ONE 4: e7186. doi:10.1371/journal.pone.0007186

14. Cleary SM, McIntyre D, Boulle AM (2006) The cost-effectiveness of antiretroviral treatment in Khayelitsha, South Africa--a primary data analysis. Cost Eff Resour Alloc 4: 20. doi:10.1186/1478-7547-4-20

**Table S[A]. Delays incorporated in the models for waiting until test results become available ("turnaround times"), by test.**

| **Turnaround times in weeks** | **India** | **South Africa** | **Uganda** |
| --- | --- | --- | --- |
| Sputum smear examination | 0 | 0 | 0 |
| Xpert MTB RIF | 0 | 0 | 0 |
| Clinical diagnosis | 0 | 0 | 0 |
| Conventional DST, solid media | 12 | N/A | 12 |
| Line-probe assay directly from sputum^1^ | N/A | 0 | N/A |
| Mycobacterial culture (liquid media) followed by line-probe assay^2^ | N/A | 4 | N/A |
| Mycobacterial culture, solid media (positive result)^3^ | 4 | N/A | 4 |
| Mycobacterial culture, solid media (negative result)^3^ | 8 | N/A | 8 |
| Mycobacterial culture, liquid media (positive result)^3^ | N/A | 2 | N/A |
| Mycobacterial culture, liquid media (negative result)^3^ | N/A | 4 | N/A |

DST: drug susceptibility testing, ^1^ smear-positive patients only, ^2^ smear-negative patients only, ^3^ sensitivity analyses only.

**Table S[B]. Treatment outcome probabilities used in the models.**

| Type of TB | Treatment regimen | Failure | | Death | | Source |
| --- | --- | --- | --- | --- | --- | --- |
|  |  |  |  |  |  |  |
| HIV-negative patients |  | probability (SE) | Distribution (α,β) | probability (SE) | distribution |  |
| New drug-suspectible | first-line, category 1 | 0.009 (0.001) | Beta (109, 11998) | 0.029 (0.001) | Beta (102, 3406) | [5] |
|  | second-line | 0.050 (0.022) | Beta (6, 97) | 0.040 (0.019) | Beta (5, 98) | [6] |
| Previously treated drug-suspectible | first-line, category 2 | 0.053 (0.009) | Beta (38, 657) | 0.048 (0.011) | Beta (18, 336) | [7] |
|  | second-line | 0.078 (0.009) | Beta (66, 770) | 0.158 (0.013) | Beta (133, 703) | [6] |
| New drug-resistant | first-line, category 1 | 0.277 (0.038) | Beta (40, 103) | 0.113 (0.027) | Beta (17, 126) | [8] |
|  | second-line | 0.050 (0.022) | Beta (6, 97) | 0.040 (0.019) | Beta (5, 98) | [6] |
| Previously treated drug-resistant | first-line, category 2 | 0.518 (0.048) | Beta (58, 54) | 0.164 (0.035) | Beta (19, 93) | [8] |
|  | second-line | 0.078 (0.009) | Beta (66, 770) | 0.158 (0.013) | Beta (133, 703) | [6] |
|  |  |  |  |  |  |  |
| HIV-positive patients |  | probability (SE) | distribution | probability (range) | distribution |  |
| New drug-suspectible | first-line, category 1 | 0.009 (0.007-0.011) | Triangular | 0.075 (0.045-0.087) | Uniform | [5] |
|  | second-line | 0.050 (0.016-0.112) | Triangular | 0.086 (0.040-0.120) | Triangular | [6] |
| Previously treated drug-suspectible | first-line, category 2 | 0.053 (0.038-0.073) | Triangular | 0.094 (0.050-0.140) | Triangular | [7][8] |
|  | second-line | 0.078 (0.061-0.098) | Triangular | 0.204 (0.100-0.300) | Triangular | [6] |
| New drug-resistant | first-line, category 1 | 0.277 (0.205-0.358) | Triangular | 0.339 (0.226-0.452) | Triangular | [8] |
|  | second-line | 0.050 (0.016-0.112) | Triangular | 0.080 (0.040-0.120) | Triangular | [6] |
| Previously treated drug-resistant | first-line, category 2 | 0.455 (0.359-0.552) | Triangular | 0.339 (0.226-0.452) | Triangular | [8] |
|  | second-line | 0.078 (0.061-0.098) | Triangular | 0.316 (0.158-0.474) | Triangular | [6] |

SE: standard error. Drug susceptible: no multidrug resistance, data based on treatment outcomes among pan-susceptible patients. Drug resistant: rifampicin resistance, assumed multidrug resistance; data based on treatment outcomes among multidrug resistant patients.

Patients with drug-susceptible TB treated with second-line regimens are assumed to have similar failure and death rates as patients with MDR-TB, separately for new and previously treated cases. All treatment outcomes adjusted for defaulting and transfer out. Failure rates for HIV-positive patients are assumed to be similar to those for HIV-negative patients, but adjusted downwards for previously treated drug-resistant cases treated with first-line category 2 regimen because distribution values for failure and cure would otherwise exceed 1. Death rates for HIV-positive are derived by adding an excess death rate of 0.046 assuming ART [9][10][11]. The mortality of HIV-infected MDR patients treated with first-line regimens is between 72 and 98% without antiretroviral treatment [12]. We assume antiretroviral treatment is given in conjunction with TB treatment, so this will lead to better survival. In studies from Thailand this resulted in about 5-fold reduction in mortality [9][10]. Survival will however be less than when second-line treatment is given, which according to Seung et al. results in 2-fold increased mortality compared to HIV- patients [13]. So we assume a 3-fold increase in mortality compared to HIV-negative patients (range 2-4). Similarly we assume a 2-fold increased death rate for HIV-infected MDR-TB patients treated with second-line regimens.

For triangular distributions the mode is represented by the probability and the lower and upper limits by the range provided.

Beta distributions have a mean
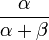
 and a variance
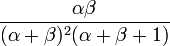
.

**Table S[C]. Probabilities used in the models of death and spontaneous recovery for undiagnosis (false-negative) untreated before a return diagnostic visit is made.**

|  | **Probability (range)** | **distribution** |
| --- | --- | --- |
| recovery, HIV-negative | 0.10 (0.085-0.115) | Triangular |
| recovery, HIV-positive | 0 (0-0.05) | Triangular |
| death, smear-positive TB, HIV-negative | 0.25 (0.213-0.288) | Triangular |
| death, smear-positive TB, HIV-positive | 1.0 (0.5-1.0) | Triangular |
| death, smear-negative TB, HIV-negative | 0.10 (0.085-0.115) | Triangular |
| death, smear-negative TB, HIV-positive | 0.67 (0.5-1.0) | Triangular |

Sourced from [1][2][3][4]

For triangular distributions the mode is represented by the probability and the lower and upper limits by the range provided.

**Table S[D]. Variables used in DALY calculations**

|  | **India** | **Uganda** | **S.Africa** | **Source** |
| --- | --- | --- | --- | --- |
| Age at onset (years) | 45 | 33 | 38 | Means from study sites |
| Life expectancy (years) | 28,1 | 33,5 | 24,1 | <http://www.who.int/whosis/database/> |
| Disability weight with TB | 0,271 | 0,271 | 0,271 | Global Burden of Disease 2004 update |
| Disability weight with HIV | 0,135 | 0,135 | 0,135 | Global Burden of Disease 2004 update |
| Disability weight with AIDS | 0,505 | 0,505 | 0,505 | Global Burden of Disease 2004 update |
| Disabiltiy weight HIV on ART | 0,167 | 0,167 | 0,167 | Global Burden of Disease 2004 update |
| Median survival untreated HIV - Sm - (years) | 7,36 | 7,36 | 7,36 | Calculated using Kaplan-Meier Survival Curve based on assumptions S3 |
| Median survival untreated HIV - Sm + (years) | 2,74 | 2,74 | 2,74 | Calculated using Kaplan-Meier Survival Curve based on assumptions S3 |
| Median survival untreated HIV+ Sm- (years) | 0,83 | 0,83 | 0,83 | Calculated using Kaplan-Meier Survival Curve based on assumptions S3 |
| Median survival untreated HIV+ Sm + (years) | 0,50 | 0,50 | 0,50 | Calculated using Kaplan-Meier Survival Curve based on assumptions S3 |
| Survival Treated TB/MDR-TB HIV + with HAART (years) | 12,9 | 12,9 | 12,9 | 12.9 additional life years associated with ART beyond HIV+ survival life expectancy [14] |
